# Development of Highly Sensitive Immunosensor for Detection of *Staphylococcus aureus* Based on AuPdPt Trimetallic Nanoparticles Functionalized Nanocomposite

**DOI:** 10.3390/mi12040446

**Published:** 2021-04-16

**Authors:** En Han, Yun Zhang, Jianrong Cai, Xinai Zhang

**Affiliations:** School of Food and Biological Engineering, Jiangsu University, Zhenjiang 212013, China; 18352764521@163.com (Y.Z.); jrcai@ujs.edu.cn (J.C.); zhangxinai@ujs.edu.cn (X.Z.)

**Keywords:** trimetallic nanoparticles, electrochemical immunosensor, label-free detection, *Staphylococcus aureus*

## Abstract

The rapid and sensitive detection of *Staphylococcus aureus* (*S. aureus*) is essential to ensure food safety and protect humans from foodborne diseases. In this study, a sensitive and facile electrochemical immunosensor using AuPdPt trimetallic nanoparticles functionalized multi-walled carbon nanotubes (MWCNTs-AuPdPt) as the signal amplification platform was designed for the label-free detection of *S. aureus*. The nanocomposite of MWCNTs-AuPdPt was prepared by an in situ growth method of loading AuPdPt trimetallic nanoparticles on the surface of MWCNTs. The synthesized MWCNTs-AuPdPt featured good conductivity and superior catalytic performance for hydrogen peroxide. The nanocomposite of MWCNTs-AuPdPt with good biocompatibility and high specific surface area was further functionalized by anti-*S. aureus* antibodies. The immobilized antibodies could efficiently capture *S. aureus* to the modified electrode by an immune reaction, which resulted in the change of catalytic current intensity to realize the sensitive detection of *S. aureus*. The designed immunosensor could detect *S. aureus* in a linear range from 1.1 × 10^2^ to 1.1 × 10^7^ CFU mL^−1^ with a low detection limit of 39 CFU mL^−1^. Additionally, the proposed immunosensor was successfully applied to determine *S. aureus* in actual samples with acceptable results. This strategy provided a promising platform for highly sensitive determination of *S. aureus* and other pathogens in food products.

## 1. Introduction

Food safety as a major public health issue has currently attracted widespread attention, which is related to ensuring healthier lives as well as protecting the national economy. *Staphylococcus aureus* (*S. aureus*), a typical and dangerous foodborne pathogen, can produce a number of toxins that cause damage to biological membranes by interacting with the host, leading to cell death [1]. *S. aureus* is recognized for its serious pathogenicity and has become an important source of many diseases, ranging from superficial skin infections to life-threatening systemic diseases such as abscesses, endocarditis, meningitis, and bacteremia [2,3,4]. Therefore, it is urgent to establish a sensitive and specific method for the detection of *S. aureus* in many fields such as food safety, medical diagnosis, and public health management.

Several methods have been developed for the detection of foodborne pathogens, including culture-based plate counting assays, polymerase chain reaction (PCR), and enzyme linked immunosensor assay (ELISA) [5,6,7]. The conventional culture methods are time-consuming and laborious, taking several days for results. Although the methods of polymerase chain reaction and enzyme-linked immunosorbent assay are powerful and exact, they need labor-intensive operation, complex sample preparation, specialized equipment, and/or the labeling of antibody, which limit their applications in real sample and rapid determination [8]. Hence, it is of great importance to develop rapid method for the detection of *S. aureus* in food samples without complex sample preparation. In recent years, the electrochemical immunosensor with a simplified setup, high sensitivity and specificity has become a new analytical platform for the detection of *S. aureus* [9,10,11]. In particular, the label-free electrochemical immunosensor is widely used on account of its simple operation and procedure [12,13,14].

Nowadays, some nanomaterials with enzymatic properties have received enormous attention due to their superiority of simple preparation, good tunability, and high stability [15,16]. Among them, noble metal nanoparticles (such as Au and Pt) have been successfully employed in the construction of electrochemical immunosensors as typical functional nanoenzymes [17]. Compared with single-metal catalysts, trimetallic nanocomposites are generally provided with more excellent properties, such as higher specific surface area and superior catalytic performance [18]. Therefore, trimetallic nanomaterials have been considered of great importance due to their unique features, opening up new horizons for improving the sensitivity of electrochemical immunosensing [19]. In particular, the combination of Au, Pd, and Pt can exhibit outstanding catalytic activity, chemical stability, and advantageous electrical conductivity [20]. More importantly, immobilization of the nanoenzyme with appropriate carbon materials can greatly increase the catalytic performance for hydrogen peroxide and provide an effective carrier for biomolecule assembly with good biocompatibility and high specific surface area [21,22,23].

In this work, a label-free electrochemical immunosensor based on AuPdPt trimetallic nanoparticles functionalized multi-walled carbon nanotubes (MWCNTs-AuPdPt) was designed for the rapid and sensitive detection of *S. aureus*. The nanocomposite of MWCNTs-AuPdPt was prepared by an in-situ growth method. The synthesized nanocomposite possessed superior electrochemical catalytic performance toward the reduction of hydrogen peroxide. The MWCNTs-AuPdPt with good biocompatibility and high specific surface area was further applied to immobilize anti-*S. aureus* antibodies to construct the electrochemical immunosensor. The immobilized antibodies could efficiently capture *S. aureus* to the modified electrode by the specific immune reaction, which resulted in the decrease of catalytic current signal. The decrease magnitude of the current intensity (Δ*I*) depended on the number of captured *S. aureus*. The proposed immunosensor achieved a wide linear range and low detection limit. Additionally, the designed immunosensor was successfully applied to the rapid detection of *S. aureus* in actual samples, providing a novel method for the detection of foodborne pathogens.

## 2. Experimental Methods

### 2.1. Reagents and Materials

Gold chloride tetrahydrate (HAuCl_4_·4H_2_O), sodium tetrachloropalladate (Na_2_PdCl_4_), platinum chloride hexahydrate (H_2_PtCl_6_·6H_2_O), L-ascorbic acid, sodium citrate, and polyvinylpyrrolidone (PVP; K-30) were bought from Shanghai Sinopharm Chemical Reagent Co., Ltd. (Shanghai, China). Bovine serum albumin (BSA) and multi-walled carbon nanotubes (MWCNTs) were purchased from Sigma-Aldrich (St. Louis, MO, USA) and Nanjing Xianfeng Nanotech Port Co., Ltd. (Nanjing, China), respectively. *S. aureus* ATCC 25923 was provided by School of Food & Biological Engineering, Jiangsu University, and stored with liquid paraffin wax at 4 °C. Anti-*S. aureus* antibody (Ab) was purchased from Abcam Inc. (Cambridge, UK). All reagents used are analytical reagent grade, and all solutions are prepared with ultra-pure water of millipore (≥18 MΩ, Milli-Q).

### 2.2. Apparatus

Transmission electron microscopy (TEM) images were obtained from JEM-2100 (JEOL Ltd., Tokyo, Japan), and scanning electron microscopy (SEM) pictures were acquired by using Quanta FEG 250 (FEI Ltd., Hillsboro, OR, USA). X-ray photoelectron spectroscopy (XPS) was recorded by AXIS-ULtra DLD (Shimadzu, Japan). All electrochemical measurements were carried out on CHI660D electrochemical workstation (Chenhua Instrument Co., Ltd., Shanghai, China). A three-electrode system was applied in all electrochemical experiments, with a glassy carbon electrodes (GCE) as the work electrode, platinum wire as the auxiliary electrode, and a saturated calomel electrode as the reference electrode.

### 2.3. Preparation of MWCNTs-AuPdPt

**Scheme 1:**
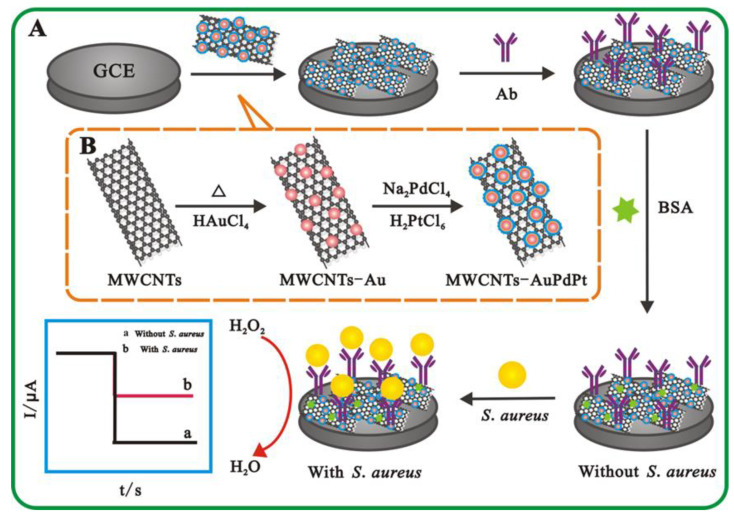
Schematic description of (**A**) the fabrication of the electrochemical immunosensor for detection of *Staphylococcus aureus* (*S. aureus*), and (**B**) the preparation procedure of multi-walled carbon nanotubes (MWCNTs)-AuPdPt.

The MWCNTs-AuPdPt nanocomposite was prepared as described in Scheme 1B. First, 20 mg of MWCNTs and 0.1 g of PVP were initially dispersed in 50 mL of HAuCl_4_ (0.3 mM) under constant stirring to obtain a homogeneous dispersion. The mixed solution was continuously boiled with vigorous stirring; then, 1 mL of trisodium citrate (1%) was immediately added to the boiling solution. Subsequently, 2 mL of ascorbic acid solution (0.1 M) was injected to the above mixture, which was followed by adding 50 mL of Na_2_PdCl_4_ solution (0.6 mM) and 50 mL of H_2_PtCl_6_ solution (0.6 mM). After being reacted for 4 min, the black hybrid solution was obtained. Finally, the product was centrifuged at 8000× *g* rpm for 10 min and washed with ultra-pure water three times; then, it was dried at 60 °C and stored at 4 °C for further use.

### 2.4. Fabrication of the Electrochemical Immunosensor

The glassy carbon electrodes (GCE) were polished with 0.3 μm and 0.05 μm Al_2_O_3_ powder on the polishing cloth. Then, GCE were washed thoroughly with HNO_3_ (1:1 *v*/*v*), absolute ethanol (95%) and ultra-pure water, and finally dried in air before use. As shown in Scheme 1A, 10 μL of MWCNTs-AuPdPt suspension was dropped to the pretreated electrode surface, which was dried at room temperature. Subsequently, 10 μL of Ab was spread onto the prepared working electrode at 4 °C for 2 h. Then, the electrode surface was rinsed completely with Phosphate-buffered saline (PBS) to remove unsuccessfully bound antibody, followed by dropping 10 μL 1% BSA solution on the electrode surface for 30 min to block non-specific reactions. Then, the electrodes were washed with PBS and stored at 4 °C until use.

### 2.5. Electrochemical Determination of S. aureus

A volume of 10 μL of suspension containing different concentration of *S. aureus* was dropped on the Ab-immobilized electrode and incubated at 37 °C for 40 min. After careful rinsing with 0.1 M PBS to remove noncaptured cells, the obtained electrode was ready for electrochemical measurement. The amperometric measurement was carried out in 0.1 M, pH 7.4 PBS at a constant potential of −0.4 V. After the background current stabilized, H_2_O_2_ solution (20 mM) was added to the buffer solution, and the changed current value at this time was recorded.

## 3. Results and Discussion

### 3.1. Characterization of MWCNTs-AuPdPt Nanocomposite

**Figure 1:**
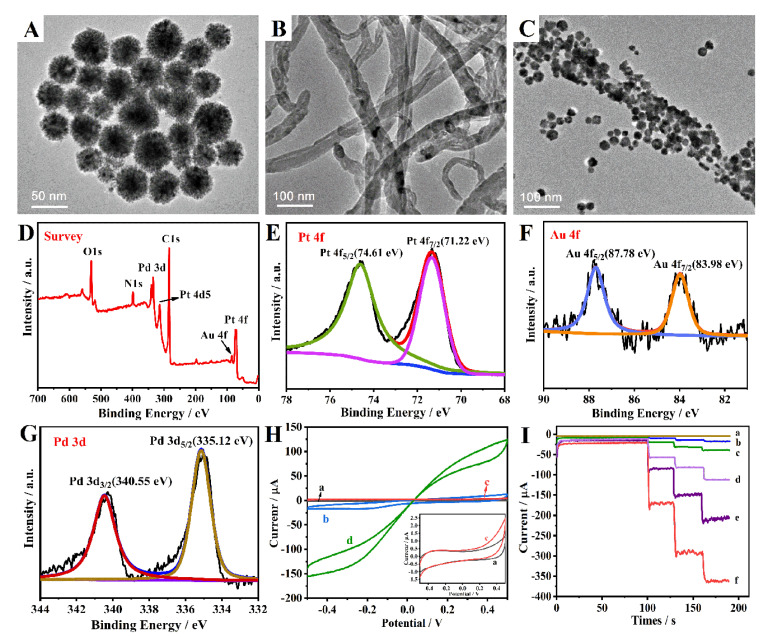
Transmission electron microscopy (TEM) images of (**A**) AuPdPt, (**B**) multi-walled carbon nanotubes (MWCNTs), (**C**) MWCNTs–AuPdPt; (**D**) X-ray photoelectron spectroscopy (XPS) spectrum of MWCNTs–AuPdPt, high-resolution XPS of (**E**) Pt 4f, (**F**) Au 4f, and (**G**) Pd 3d of the MWCNTs-AuPdPt; (**H**) cyclic voltammetry (CV) curves of (a) glassy carbon electrodes (GCE) and (b) GCE/MWCNTs-AuPdPt in Phosphate-buffered saline (PBS) solution, and (c) GCE and (d) GCE/MWCNTs–AuPdPt in PBS solution containing 10 mM H_2_O_2_; (**I**) Ameperometic responses of different modified electrodes for the successive addition of 10 mM H_2_O_2_ in PBS solution: (a) GCE, (b) GCE/MWCNTs, (c) GCE/Au, (d) GCE/AuPd, (e) GCE/AuPdPt, and (f) GCE/MWCNTs-AuPdPt. Inset in Figure 1H: the enlarged CVs of curve (a) and (c).

The structure and morphology of the nanocomposite was firstly examined by TEM imaging. Figure 1A illustrated the nanoflower structure of AuPdPt synthesized individually with a mean size of 50 nm. As shown in Figure 1B, MWCNTs had the characteristics of smooth surface and good dispersion, and the average diameter of MWCNTs was about 50 nm. Compared with MWCNTs, Figure 1C showed that numerous AuPdPt trimetallic nanoparticles were universally assembled on the surface of MWCNTs by the in situ growth method, which could be clearly distinguished from AuPdPt and MWCNTs. XPS was further introduced to analyze the elemental composition of MWCNTs-AuPdPt. As shown in Figure 1D, the XPS survey spectrum showed characteristic peaks for C, O, N, Pt, Au, and Pd. These elements could be further analyzed and determined by peak fitting. According to Figure 1E, the Pt 4f spectrum exhibited two characteristic peaks at 74.61 eV and 71.22 eV, which were attributed to Pt 4f5/2 and Pt 4f7/2, respectively. From Figure 1F, the peaks of Au 4f5/2 and Au 4f7/2 could be seen at 87.78 eV and 83.98 eV, denoting the successful formation of Au in the nanocomposite. The high-resolution spectrum of Pd 3d displayed double peaks at 340.55 eV and 335.12 eV, corresponding to the Pd 3d3/2 and Pd 3d5/2, respectively (Figure 1G). The above results were in agreement with previous reports [24,25]. These results further indicated that AuPdPt trimetallic nanoparticles were successfully modified on the surface of MWCNTs.

Due to the synergistic effect of AuPdPt nanoparticles and the MWCNTs, the synthesized MWCNTs-AuPdPt nanozyme was found to have enhanced peroxidase performance. As shown in Figure 1H, the cyclic voltammetry (CV) of GCE/MWCNTs-AuPdPt in the absence of H_2_O_2_ manifested a small current response (curve b), which was approximately 20 μA higher than the background current (curve a). When 10 mM H_2_O_2_ was added to the PBS, the detection signal increased dramatically (curve d), and its current signal increased by about 130 μA compared with curve b. Hence, it revealed that MWCNTs-AuPdPt could be employed as excellent nanocomposites to construct a label-free electrochemical immunosensor with high sensitivity and realize the amplification of the detection signal of the immunosensor.

In order to compare the catalytic performance of different modified electrodes, the experiment further investigated the amperometric responses of bare GCE, GCE/MWCNTs, GCE/Au, GCE/AuPd, GCE/AuPdPt, and GCE/MWCNTs-AuPdPt with the continuous addition of 10 mM H_2_O_2_. As represented in Figure 1I, bare GCE had no catalytic effect on the reduction of H_2_O_2_ (curve a), but the current signal slightly improved upon MWCNTs that were modified on the electrode (curve b), which was attributed to the intrinsic peroxidase-like activity of carbon nanotubes [26]. Compared with curve a, b, c and d, GCE/AuPdPt (curve e) exhibited good current response on account of the excellent properties of the combination of trimetallic nanocomposites. After MWCNTs-AuPdPt was loaded on the electrode, a significantly increased current signal could be observed (curve f), which was ascribed to the synergistic effect of AuPdPt and MWCNTs.

### 3.2. Characterization of the Electrochemical Immunosensor

**Figure 2:**
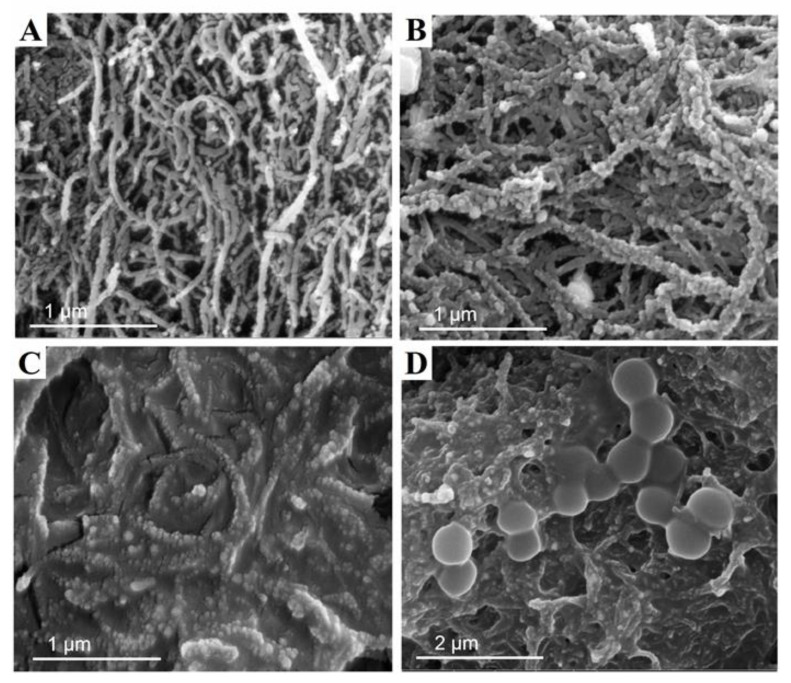
Scanning electron microscopy (SEM) images of (**A**) MWCNTs, (**B**) MWCNTs-AuPdPt, (**C**) MWCNTs-AuPdPt/Anti-*S. aureus* antibody (Ab), and (**D**) MWCNTs-AuPdPt/Ab/*Staphylococcus aureus* (*S. aureus*).

SEM imaging was employed to characterize the morphologies of the different modified electrodes in the process of immunosensor fabrication. As shown in Figure 2A, the MWCNTs with diameters of 50–70 nm displayed a well-dispersed structure in the form of small bundles. Compared with MWCNTs, a denser homogeneous structure with a large number of AuPdPt nanoparticles could be observed for the MWCNTs-AuPdPt nanocomposite film (Figure 2B), indicating the successful preparation of MWCNTs-AuPdPt nanocomposites. As shown in Figure 2C, after the anti-*S. aureus* antibody was immobilized onto the MWCNTs-AuPdPt film, the tube structure became plumper, and the structure of film was completely changed, confirming that the anti-*S. aureus* antibody was bound successfully. This three-dimensional nanostructure was in favor of the recognition reaction between the antibody and surface antigen of *S. aureus*. After the immune reaction between *S. aureus* and the *anti-S. aureus* antibody functionalized on the electrode, the cells of *S. aureus* were largely captured onto the functional electrode (Figure 2D), indicating that the design of immunosensor based on MWCNTs-AuPdPt was feasible.

### 3.3. Optimization of Experimental Conditions

**Figure 3:**
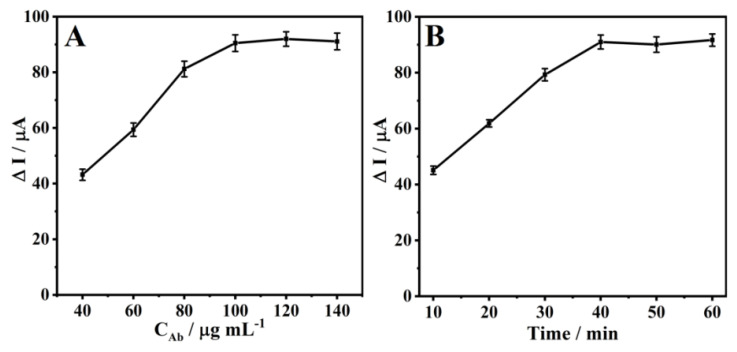
Optimization of the experimental conditions of (**A**) Ab concentration and (**B**) incubation time of *S. aureus* (1.2 × 10^5^ colony-forming units (CFU)/mL).

In order to obtain excellent analytical performance of the immunosensor, some important parameters such as concentration of antibody and incubation time of *S. aureus* were investigated. The concentration of *S. aureus* for optimization was 1.2 × 10^5^ colony-forming units (CFU)/mL. When one parameter changed, the other parameter was at the optimal value. CFU is a measure of viable bacteria or fungal cell numbers in CFU/mL. It is a way to calculate the number of microorganisms in a sample based on the number of colonies visible to the naked eye on growth medium. The amount of anti-*S. aureus* antibody on the surface of electrode had a significant effect on the current signal of the electrochemical immunosensor. Therefore, the concentration of the anti*-S. aureus* antibody used for the capture of *S. aureus* was investigated. As shown in Figure 3A, the Δ*I* value increased with the increasing concentration of antibody from 40 to 100 μg mL^−1^, and it did not change significantly at the concentration of 100 to 140 μg mL^−1^. This phenomenon may be attributed to the fact that the more sites recognized by *S. aureus* with the increase of antibody concentration, but when the antibody concentration was higher than 100 μg mL^−1^, the antibody assembled on the electrode had reached saturation. The optimal concentration of anti-*S. aureus* antibody was taken as the lowest concentration that yielded saturated cell-binding densities. Therefore, 100 μg mL^−1^ was selected as the optimal antibody concentration for the detection of *S. aureus*. The effect of the incubation time for the immune reaction between anti-*S. aureus* antibody and *S. aureus* was explored within 10 to 60 min. As shown in Figure 3B, the Δ*I* value increased when the reaction time increased from 10 to 40 min, and then, it remained unchanged when the incubation time was more than 40 min, indicating the sufficient binding of anti-*S. aureus* antibody and *S. aureus*. Thus, 40 min was chosen as the optimal immune reaction time.

### 3.4. Electrochemical Immunosensor Detection of S. aureus

**Figure 4:**
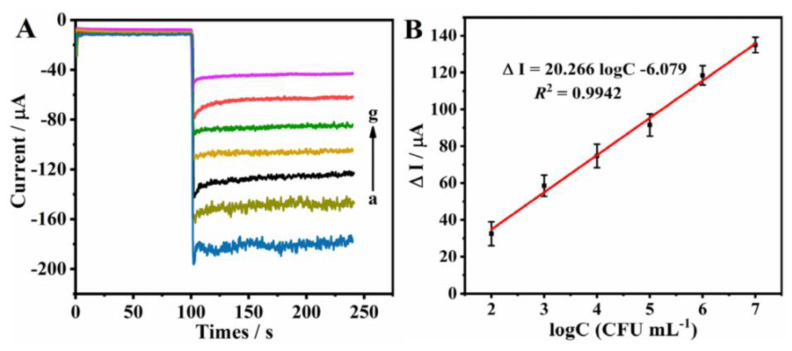
**(A)** Ameperometic current responses of immunosensor for the detection of different concentrations of *S. aureus* from a to f (0, 1.1 × 10^2^, 1.1 × 10^3^, 1.1 × 10^4^, 1.1 × 10^5^, 1.1 × 10^6^, 1.1 × 10^7^ CFU mL^−1^) in 0.1 M PBS containing 20 mM H_2_O_2_ at −0.4 V, and (**B**) plots of Δ*I* values vs. logarithm value of the concentrations of *S. aureus*.

Under the optimal condition, the immunosensor exhibited sensitive response to *S. aureus* in 0.1 M PBS containing 20 mM H_2_O_2_ based on MWCNTs-AuPdPt nanoenzyme. The concentration of 20 mM of H_2_O_2_ was obtained by optimization (not shown here). As shown in Figure 4A, the amperometric response of the electrochemical immunosensor significantly declined with the increase of the concentration of *S. aureus*. This should be due to the fact that with the increase of *S. aureus* concentration, the immune complex formed on the sensing interface enhanced the effect of hindering electron transport, thus reducing the current signal [27]. The calibration curve showed a linear relationship between the Δ*I* value and the logarithm of *S. aureus* concentration in the range from 1.1 × 10^2^ to 1.1 × 10^7^ CFU mL^−1^ with a low limit of detection of 39 CFU mL^−1^ (Figure 4B). The linear regression equation of the obtained calibration curve was Δ*I* = 20.266logC − 6.079 with a correlation coefficient of 0.994. The limit of detection (LOD) for *S. aureus* was calculated according to the report based on three times the standard deviation divided by the slope of the calibration curve [28]. Furthermore, the analytical performance of the proposed immunosensor for the detection of *S. aureus* was compared with other reports (Table 1). It can be inferred that the fabricated immunosensor in this work exhibited a wider detection range and a lower detection limit. In summary, the label-free electrochemical immunosensor provided a prospective method for the high sensitivity determination of *S. aureus*, which may be ascribed to the following two factors: (1) The nanocomposite of MWCNTs-AuPdPt with good biocompatibility and high specific surface area provided an excellent platform for loading biomolecules; and (2) the synergistic effect of the prepared MWCNTs-AuPdPt nanozyme showed excellent electrochemical performance and enhanced catalytic activity for H_2_O_2_.

### 3.5. Reproducibility, Stability, and Specificity of the Immunosensor

**Figure 5:**
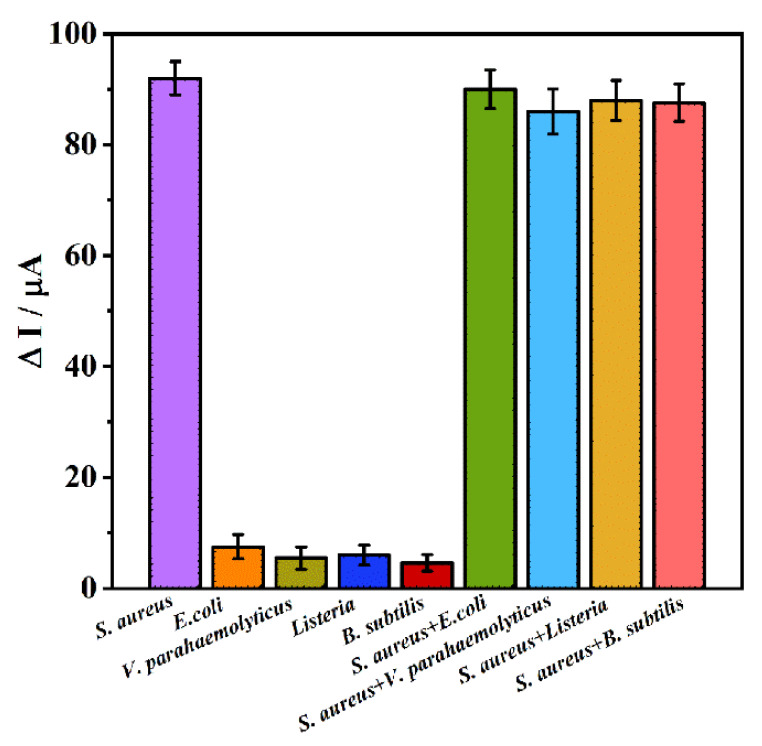
Specificity investigation of the immunosensor to different microorganisms. The concentration of the interfering bacteria is 2.4 × 10^5^ CFU mL^−1^.

The specificity of the immunosensor was analyzed by adding the same concentration of interfering bacteria such as *E. coli*, *V. parahaemolyticus*, *L. monocytogenes,* and *B. subtilis* (2.4 × 10^5^ CFU mL^−1^). As shown in Figure 5, the Δ*I* value of *S. aureus* was considerably higher than that of other interference groups. It was worthwhile that when the immunosensor was used to detect the mixture of *S. aureus* (1.8 × 10^5^ CFU mL^−1^) and interfering bacteria (2.4 × 10^5^ CFU mL^−1^), the Δ*I* value was increased obviously, indicating that the immunosensor was highly selective for the detection of *S. aureus*.

To examine the reproducibility of the designed immunosensor, eight different electrodes were prepared at the same time to fabricate immunosensors for the detection of 1.5 × 10^4^ CFU mL^−1^ of *S. aureus*. The relative standard deviation (RSD) of the detection results for the eight electrodes was found to be 6.3%, suggesting the good reproducibility of the immunosensor. In addition, the stability of the developed immunosensor was also studied for the detection of 1.5 × 10^4^ CFU mL^−1^ of *S. aureus*. When the immunosensor was stored at 4 °C in a refrigerator, the current response still remained at 91% of the original response after a storage period of 3 weeks. The experimental result denoted that the stability of the immunosensor was acceptable.

### 3.6. Determination of *S. aureus* in Real Samples

In order to demonstrate the potential application of this immunosensor in the analysis of actual samples, the designed immunosensor was applied in the detection of *S. aureus* spiked in three kinds of samples (yogurt, pure milk, and milk powder) with a standard addition method. Pure milk was the boxed milk purchased from the local supermarket. Milk powder sample was first dissolved in PBS; then, it was heated and stirred until it was completely dissolved, while the other two samples did not need further treatment except for the dilution step. These analyses were performed for three times under the same conditions, and the results listed in Table 2 were also compared with the traditional plate count method. The recoveries of standard additions for *S. aureus* in the spiked samples were in the range of 91.2–103.0% with the RSD of 4.9–7.5%. The above results demonstrated that the developed biosensor was provided with high accuracy and satisfactory application potential, and it could be employed for the detection of *S. aureus* in the field of food monitoring.

## 4. Conclusions

A novel electrochemical immunosensor has been successfully fabricated for the label-free and highly sensitive determination of *S. aureus* by using the MWCNTs-AuPdPt nanocomposite. The nanocomposite of MWCNTs-AuPdPt prepared by an in situ growth method combines the two advantages of AuPdPt nanoparticles and MWCNTs, which include good conductivity, high specific surface area for loading protein molecules, and superior catalytic performance toward the reduction of hydrogen peroxide. The developed electrochemical immunosensor for assay of *S. aureus* with a broad linear range and a low detection limit has been demonstrated to possess perfect specificity, high sensitivity, and acceptable reproducibility. Additionally, this novel electrochemical immunosensor is successfully applied to determine *S. aureus* in food samples without complicated sample pretreatment. Therefore, this strategy is of great significance for the rapid and highly sensitive detection of *S. aureus* in actual food samples.

## Figures and Tables

**Table 1:** Comparison of the designed immunosensor for the detection of *Staphylococcus aureus* (*S. aureus*) with other studies in the literature.

Substrate of Immunosensors	Linear Range (CFU mL^−1^)	Limit of Detection (LOD) ^3^ (CFU mL^−1^)	Ref.
Ag–MnO_2_	10^3^ to 10^7^	-	[29]
Magnetic–silica particles	8.0 × 10^2^ to 1.0 × 10^4^	6.8 × 10^2^	[30]
Au/Pt NCs ^1^	10^2^ to 10^8^	80	[31]
AgNPs/3D-ZnO nanorods	10^3^ to 2 × 10^3^	3.3 × 10^2^	[32]
Gold nanodisks	10^3^ to 10^8^	10^3^	[33]
VAN ^2^-Au NPs	10^3^ to 10^8^	10^3^	[34]
MWCNTs-AuPdPt	1.1 × 10^2^ to 1.1 × 10^7^	39	This work

^1^ NCs: nanoclusters; ^2^ VAN: vancomycin; ^3^ LOD: limit of detection.

**Table 2:** Determination of *S. aureus* in real samples by using the immunosensor and plate count method (*n* = 3).

Samples	Spiked (CFU mL^−1^)	Found (CFU mL^−1^)	Plate Count (CFU mL^−1^)	RSD (%)	Recovery (%)
Yogurt	2.32 × 10^3^	2.39 × 10^3^	2.36 × 10^3^	6.1	103.0
	2.32 × 10^4^	2.19 × 10^4^	2.28 × 10^4^	7.5	94.4
	2.32 × 10^5^	2.23 × 10^5^	2.33 × 10^5^	6.2	96.1
Pure milk	3.41 × 10^3^	3.45 × 10^3^	3.48 × 10^3^	5.6	101.2
	3.41 × 10^4^	3.26 × 10^4^	3.36 × 10^4^	4.9	95.6
	3.41 × 10^5^	3.30 × 10^5^	3.43 × 10^5^	5.8	96.8
Infant milk powder	2.95 × 10^3^	2.69 × 10^3^	3.05 × 10^3^	6.8	91.2
	2.95 × 10^4^	2.85 × 10^4^	2.88 × 10^4^	5.9	96.6
	2.95 × 10^5^	2.73 × 10^5^	2.92 × 10^5^	7.1	92.5
